# Dystrophic Changes in Extraocular Muscles after Gamma Irradiation in *mdx:utrophin^+/−^* Mice

**DOI:** 10.1371/journal.pone.0086424

**Published:** 2014-01-21

**Authors:** Abby A. McDonald, Matthew D. Kunz, Linda K. McLoon

**Affiliations:** Department of Ophthalmology and Visual Neurosciences, and Graduate Program in Molecular, Cellular, Developmental Biology and Genetics, University of Minnesota, Minneapolis, Minnesota, United States of America; University of Pittsburgh, United States of America

## Abstract

Extraocular muscles (EOM) have a strikingly different disease profile than limb skeletal muscles. It has long been known that they are spared in Duchenne (DMD) and other forms of muscular dystrophy. Despite many studies, the cause for this sparing is not understood. We have proposed that differences in myogenic precursor cell properties in EOM maintain normal morphology over the lifetime of individuals with DMD due to either greater proliferative potential or greater resistance to injury. This hypothesis was tested by exposing wild type and *mdx:utrophin^+/−^* (*het*) mouse EOM and limb skeletal muscles to 18 Gy gamma irradiation, a dose known to inhibit satellite cell proliferation in limb muscles. As expected, over time *het* limb skeletal muscles displayed reduced central nucleation mirrored by a reduction in Pax7-positive cells, demonstrating a significant loss in regenerative potential. In contrast, in the first month post-irradiation in the *het* EOM, myofiber cross-sectional areas first decreased, then increased, but ultimately returned to normal compared to non-irradiated *het* EOM. Central nucleation significantly increased in the first post-irradiation month, resembling the dystrophic limb phenotype. This correlated with decreased EECD34 stem cells and a concomitant increase and subsequent return to normalcy of both Pax7 and Pitx2-positive cell density. By two months, normal *het* EOM morphology returned. It appears that irradiation disrupts the normal method of EOM remodeling, which react paradoxically to produce increased numbers of myogenic precursor cells. This suggests that the EOM contain myogenic precursor cell types resistant to 18 Gy gamma irradiation, allowing return to normal morphology 2 months post-irradiation. This supports our hypothesis that ongoing proliferation of specialized regenerative populations in the *het* EOM actively maintains normal EOM morphology in DMD. Ongoing studies are working to define the differences in the myogenic precursor cells in EOM as well as the cellular milieu in which they reside.

## Introduction

Skeletal muscle has a remarkable capacity for regeneration after injury and in disease. The process of regeneration is dependent on a population of myogenic precursor cells that were originally defined by their position between the sarcolemma and basal lamina and called satellite cells [1]. These cells are normally quiescent. Upon injury, these myogenic precursor cells are activated and begin a process of proliferation and self-renewal. Their progeny can either fuse into injured fibers or fuse together to form new myofibers [2]. One generally accepted cellular marker for satellite cells is Pax7, which labels these cells when they are quiescent and is down-regulated when the cells become activated and begin to express MyoD [3], [4]. In diseases like Duchenne muscular dystrophy (DMD) and in age-related sarcopenia, the regenerative capacity of limb and body skeletal muscle becomes exhausted primarily due to exhaustion of the satellite cell pool after repeated cycles of muscle degeneration and regeneration, ultimately leading to a loss of muscle mass and function [5], [6], [7], [8], [9]. Not all skeletal muscles are equally affected by DMD, and notable exceptions exist that appear to be completely spared in DMD and related muscular dystrophies. Spared skeletal muscles include a number of craniofacial muscles, including the laryngeal and extraocular muscles [10], [11].

The extraocular muscles (EOM) are craniofacial muscles responsible for the complex and finely controlled movements of the eyes. While they are skeletal muscles, the EOM are considered a distinct allotype, with a number of differences that distinguish them from limb skeletal muscles [12]. In muscular dystrophies such as DMD, the EOM are not only morphologically spared, but are functionally spared as well [11], [13]. While many causes for the sparing of EOM in DMD have been proposed, none have proven mechanistic [14], [15], [16]. It is currently thought that there are constitutive differences between the EOM and limb skeletal muscles that account for their preferential sparing [17].

Unlike adult non-craniofacial skeletal muscle, normal uninjured adult EOM maintain continuously activated satellite cells, allowing the EOM to continuously remodel throughout life [18], [19], [20], even in the EOM from elderly humans [21]. Persistently activated satellite cells may be due to a unique and/or more abundant subpopulation of myogenic precursor cells retained within the adult EOM. This population of cells also may have different capacities, such as enhanced survival or greater proliferative capacity, which allow the EOM to continuously remodel without stem cell exhaustion, different than what is seen in limb skeletal muscle [5]. In addition to maintaining this population of activated myogenic precursor cells, when subjected to various types of muscle injury or drug treatments, EOM and limb muscles tend to respond in significantly different ways. One example is the response of these two groups of skeletal muscles to chemical denervation with botulinum toxin A injections. In treated EOM, the satellite cells begin to proliferate robustly, and muscle mass is maintained [22]. In contrast, in limb skeletal muscle botulinum toxin injection produces only an abortive regenerative response and is followed by muscle atrophy.

One candidate population enriched in the EOM compared to limb skeletal muscle that we previously identified is the EECD34 cell population, which is CD34^+^/Sca1^−/^CD31^−/^CD45^−^ [23], [24]. The EECD34 cells are myogenic and are maintained in aging EOM. In mouse models of DMD, the population of EECD34 cells declines precipitously in limb skeletal muscle but is preferentially retained in the EOM. Interestingly, the EECD34 cells derived from the EOM have increased proliferative capacity even compared to the “same” cells derived from limb skeletal muscle [24]. Recently we have shown a subpopulation of myogenic precursor cells that is distinct from the Pax7-positive cells that are up-regulated in EOM compared to limb skeletal muscle. These cells express Pitx2 and are also retained preferentially in the EOM of mouse models of muscular dystrophy and in aging skeletal muscle.

In order to determine if sparing of the EOM in DMD might be due to a greater proliferative capacity and/or a greater resistance to injury of one or more of its populations of myogenic precursor cells, including Pax7, EECD34, and Pitx2-positive cells, we designed gamma irradiation experiments in order to inhibit the proliferation of myogenic precursor cells. Irradiation has been frequently used to inhibit myogenic precursor cell proliferation and regeneration in limb skeletal muscles, including those of dystrophic mice [25], [26], [27], [29], [30], [31], [32], in order to assess the importance of regeneration in skeletal muscle disease and pathology.

Wild type (C57BL/10) (WT) and dystrophic *mdx:utrophin^+/−^* (*het*) mice were treated with an 18 Gy dose of gamma irradiation, targeting both EOM and limb skeletal muscle. This mouse genotype was chosen for a number of reasons. First, these *mdx* mice haploinsufficient for utrophin have a pathology that more closely mimics human DMD than the *mdx* mouse [33]. The *mdx:utrophin^−/−^* (*dKO*) mouse model manifests a very severe skeletal muscle pathology [34], and recent work shows that in addition to severe muscle degeneration and regeneration-deficient muscle stem cells, they also suffer from pathologies of bone and cartilage [35]. Muscles from WT and *het* mice were collected at various post-irradiation end-points and assessed for morphological characteristics, in order to determine if inhibition of proliferation by gamma irradiation would affect disease pathology in EOM and limb skeletal muscles and whether this would result in loss of the myogenic precursor cells needed for maintenance of the normal phenotype in the EOM of the *het* mice. If greater proliferative capacity of one or more of the populations of myogenic precursor cells is responsible for maintaining a normal EOM phenotype, even in skeletal muscle disease, then inhibiting proliferation should result in the appearance in the EOM of pathology characteristic of dystrophic limb muscles.

## Methods

### Animal Care

All experiments were approved by the Institutional Animal Care and Usage Committee at the University of Minnesota and performed in accordance with NIH guidelines for use of animals in research. All mice used in these experiments were maintained by Research Animal Resources at the University of Minnesota. Mice were raised in 12-hour light/dark cycles and were allowed to feed and drink *ad libitum*. C57BL/10 mice (Harlan, Indianapolis, IN) were used as wild type (WT) controls. Dystrophic mice (*mdx:utrophin^+/−^ [het]*) were maintained as a colony at the University of Minnesota through *mdx:utrophin^+/−^* breeding pairs that originated from Washington University (ECR 42). Four to eight mice were used per experiment. The *mdx:utrophin^+/−^* mouse line was chosen specifically for its pattern of skeletal muscle pathology relative to other genotypes. It is well known that the muscle pathology of the mdx mouse does not closely mimic human DMD, as the mice have a normal lifespan. They display a significant degenerative/regenerative response starting at approximately 1 month but the pathology is significantly reduced by 6 months [36], [37]. Thus we worried that the return to a more normal phenotype in the limb muscles of these mdx mice would mask the effects of irradiation. Similarly the *mdx:utrophin^−/−^* mouse is extremely sick [34], [38]. In our hands, these mice did not survive longer than 1–2 months, which was too short for the long post-irradiation intervals required for this study. Our first attempts at irradiation in these mice showed them to be extremely fragile relative to anesthesia, even when closely monitored. Several articles were published in the last few years indicating that the *mdx:utrophin^+/−^* mouse has a phenotype that more closely mimics the disease course in DMD patients [33], [39]. Thus, this mouse genotype was chosen for our experimental manipulations.

### Gamma Irradiation

Both *het* and WT mice were irradiated at a single dose of 18 Gray (Gy) [40]. Mice were first anesthetized with a ketamine:xylazine cocktail (100 mg/ml, 10∶1, 1.1 mL/kg weight). Anesthetized mice were placed in a JL Shepard irradiator (^137^Cs source), and entirely shielded with lead excluding the anterior face region, including the orbits, and the right forelimb. Once properly shielded, the mice received a dose of 18 Gy irradiation and removed from the irradiator. Mice were observed until they regained consciousness. Mice were euthanized either for histochemistry or flow cytometry at the designated end points. Non-irradiated animals served as controls for both WT and *het* mice.

### Immunohistochemistry

At selected post-irradiation time points, mice were euthanized by tank carbon dioxide inhalation, and immediately following sacrifice an orbital exenteration was performed removing the globe with extraocular muscles (EOM) attached *in situ* and triceps muscles of the left and right forelimb were dissected, removed, embedded in tragacanth gum, and frozen in 2-methylbutane chilled to a slurry on liquid nitrogen. Sections were prepared at 12 µm using a cryostat and stored at −30°C until stained. One set of sections was stained with hematoxylin and eosin. Additional sections were immunostained with antibodies to collagen I and IV (1∶1000, Abcam, Cambridge, MA). Briefly, after blocking in normal serum, selected sections were incubated in primary antibody for 1 hour. The sections were rinsed in phosphate buffered saline (PBS), followed by incubation using reagents in the Vectastain Elite ABC kit (Vector Laboratories, Burlingame, CA). Visualization was obtained by incubation in diaminobenzidine using heavy metal intensification and hydrogen peroxide. Specificity of antibody binding was verified by processing sections in the absence of primary antibody. Satellite cells were immunostained with Pax7 (1∶3000, Aviva Systems Biology, San Diego, CA) using the procedure outlined for collagen.

To visualize Pitx2-positive myogenic precursor cells tissue sections were blocked in 20% goat serum/0.2% BSA in phosphate buffered saline containing 0.1% triton X-100 (antibody buffer) for 30 minutes at room temperature, incubated with an antibody to Pitx2 (Capra Science, 1∶2000) in antibody buffer overnight at 4°C, rinsed, blocked for 10 minutes in 20% goat serum/0.2% BSA in antibody buffer, and incubated with goat anti-rabbit-AlexaFluor 488 antibody (Jackson ImmunoResearch, 1∶2000) in antibody buffer for 1 hour at room temperature [24]. After a PBS rinse, sections were incubated for 15 minutes with wheat germ agglutinin (WGA) conjugated to Texas Red X (Life Technologies, Eugene, OR, 1∶500) at room temperature. Sections were rinsed, coverslipped, and examined using epifluorescence microscopy. A subset of sections were immunostained for Pitx2 using an anti-rabbit Rhodamine Red (1∶500), followed by brdU immunostaining using donkey anti-mouse Alexa Fluor 488 (Jackson ImmunoResearch).

### Analysis

For central nucleation, a hallmark of cycles of degeneration and regeneration, data is presented as percent of centrally nucleated myofibers per total fiber number averaged for several fields/section. A minimum of 200 myofibers were analyzed in random fields in three muscle cross-sections per animal and for each of the two layers of EOM and averaged. A total of 6 mice were used for each time point analyzed.

Pax7-positive and Pitx2-positive nuclei were counted based on myofiber number, with a minimum of 200 fibers counted per muscle section in random fields for leg muscles and for each layer in the EOM. Density of Pax7 or Pitx2-positive cells per myofiber number per muscle section was determined from 3–4 fields per muscle section, 3 muscle sections averaged per mouse, and averaged for six mice at each of the experimental end points examined.

Collagen density was determined using the automated morphometry feature of the Bioquant Image Analysis System (Bioquant, Nashville, TN). The area of each microscopic field positive for each specific collagen was determined, and the total collagen-positive area was calculated based on the total muscle area per field, with 3–4 fields chosen per muscle section, 3 muscle sections analyzed and averaged per mouse, with an N of 6 mice per experimental time point and genotype.

### Bromodeoxyuridine Labeling

To assess the effectiveness of 18 Gy irradiation on satellite cell division, bromodeoxyuridine (brdU) labeling was performed using our standard method [18], [20]. Briefly, all mice received intraperitoneal injections of bromodeoxyuridine in sterile isotonic saline once per day for 7 days at a dose of 50 mg/kg body weight. Sections were quenched with hydrogen peroxide and incubated sequentially with blocking serum and biotin-avidin blocking reagent (Vector Laboratories). After a rinse in PBS, sections were incubated in 2N HCl for 1 hour at 37°C, followed by neutralization in borate buffer and a PBS rinse. The sections were incubated in the primary antibody to brdU for 1 hour at room temperature (Roche, Indianapolis, IN; diluted 1∶1000). After a PBS rinse, the sections were incubated using the reagents in the Vectastain peroxidase kit (Vector Laboratories), and reacted with diaminobenzidine containing heavy metals and hydrogen peroxide.

### Flow Cytometry

At the selected post-irradiation time points, mice were euthanized by tank carbon dioxide inhalation, and immediately following sacrifice, the EOM and the muscles of the right forelimb were isolated into Dulbecco’s modified Eagle’s medium (DMEM) (Invitrogen, Carlsbad, CA). Mononuclear cells were prepared from the muscles by mincing leg muscle into small pieces; EOM did not require mincing due to their small size. All tissue was digested with collagenase type B and dispase type II (Roche Diagnostics, Indianapolis, IN) at 37°C for 15 minutes, triturated, and incubated for another 15 minutes until no visible chunks of tissue remained. Following digestion, the cell suspensions were filtered through a 70 µm nylon cell strainer to remove debris. Cells were then washed with sorter buffer, which was prepared with phosphate-buffered saline (PBS) (Invitrogen) containing 25 mM HEPES, 2 mM EDTA, and 1% fetal calf serum (Atlanta Biologicals, Lawrenceville, GA). Cells were resuspended in sorter buffer and stained with the following antibodies: Sca1, CD34, CD31, and CD45 (BD Biosciences, San Jose CA) [24]. Cells were incubated with antibodies for 30 minutes at 4°C and washed with sorter buffer. To exclude dead and dying cells from analysis, 7-aminoactinomycin D (7AAD) (BD Biosciences) was added at a 1∶25 dilution 10 minutes before flow cytometry analysis. Cells were analyzed on a FACS Canto (BD Biosciences) housed in the Stem Cell Institute at the University of Minnesota, and final analysis was performed using the FlowJo software (Tree Star, Inc., Ashland, OR ). All analyses were based on a total of 5 independent muscle isolations from different mice per experimental time point.

### Statistical Analysis

Data was expressed as mean +/− SEM. All data were analyzed for statistical significance using an analysis of variance (ANOVA) followed by either a Tukey’s or Dunn’s multiple comparison test aided by the Prism and Statmate software (GraphPad Software Inc., San Diego, CA) for multiple group comparisons. Data were considered statistically significantly different if p<0.05.

## Results

### Gamma Irradiation of Wild Type and Het Mice Limb Muscles

**Figure 1:**
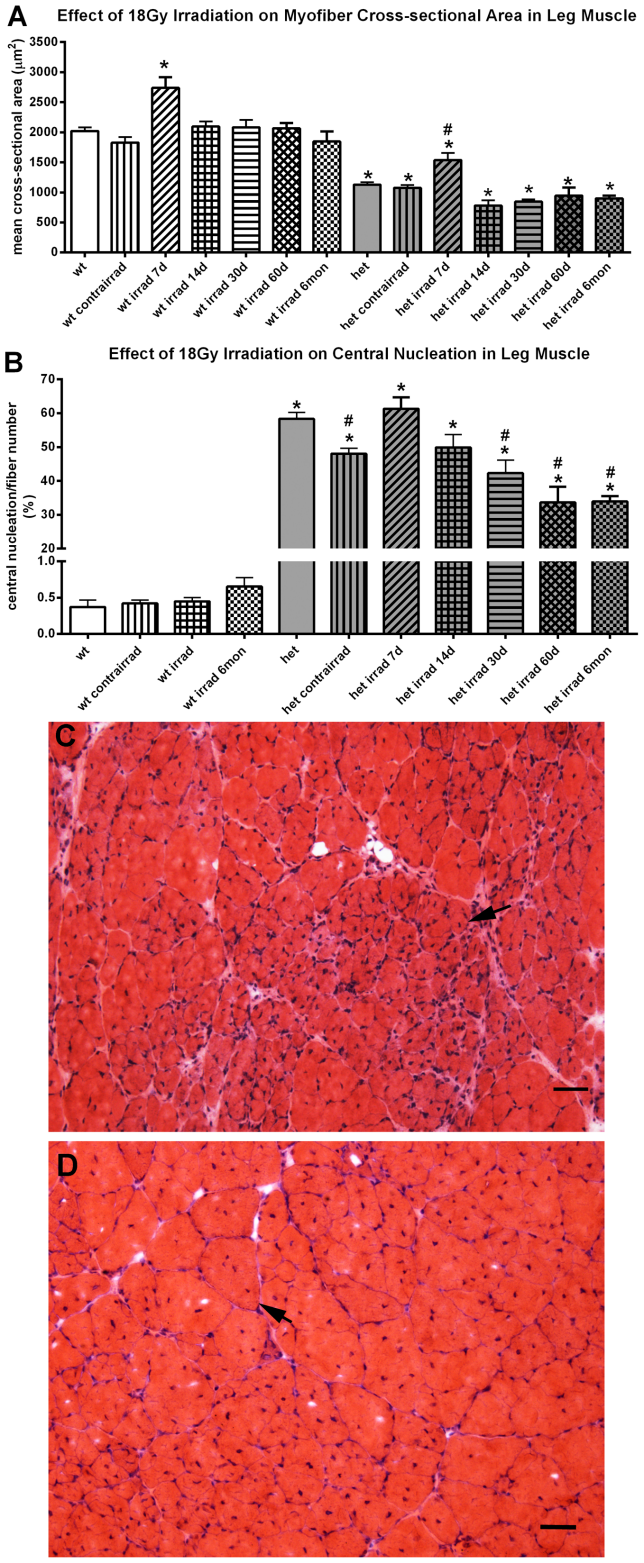
Effect of 18 Gy Irradiation on Cross-sectional Areas and Central Nucleation in Limb Muscles from Wild Type and *Het* Mice. A. Effect of 18 Gy irradiation on cross-sectional areas in leg muscle from wild type (wt) and *mdx:utrophin^+/−^* mice (*het*). *indicates significant difference from non-irradiated WT control. # indicates significant difference from non-irradiated *het* control. B. Effect of 18 Gy irradiation on central nucleation in leg muscle from WT and *het* mice. *indicates significant difference from non-irradiated *WT* control. # indicates significant difference from 18 Gy 7 day and 14 day *het* leg muscle. C. Photomicrograph of *het* leg muscle 60 days after gamma irradiation at 18 Gy. Arrow indicates a large group of very small myofibers. D. Photomicrograph of non-irradiated age-matched *het* leg muscle. Arrow indicates a myofiber with multiple centrally located myonuclei. Bar is 20 µm.

As predicted from the literature and as visualized with hematoxylin and eosin, no overt histological alteration was seen in the 18 Gy irradiated limb muscle from WT mice. No inflammatory infiltrate or myofiber necrosis was seen at any of the post-irradiation time points (not shown). The cross-sectional area of WT irradiated mouse limb muscle increased transiently at one week post-irradiation by approximately 38% compared to non-irradiated WT controls, but then returned to normal and did not change from 2 weeks up to six months after the 18 Gy irradiation (Figure 1A). Central nucleation was examined in order to assess the process of degeneration/regeneration in these muscles. After 18 Gy treatment of WT mouse limb muscles, no change in centralized myonuclei density was seen (Figure 1B).

As seen in the WT irradiated limb muscle, no evidence of increased inflammatory infiltrate or widespread myofiber necrosis was seen in the irradiated *het* limb muscles (Figure 1 C, D). Note that the shielded limb muscle contralateral to the irradiated foreleg was not significantly different from the non-irradiated *het* muscles (Figure 1A). However, compared to the WT mice, the limb muscles were significantly affected by gamma irradiation in the *het* mouse (Figure 1). The mean cross-sectional area of the untreated *het* triceps myofibers was 43.9% smaller than the normal control myofibers, which was highly significant. As seen in the WT control triceps muscle, there was a transient 36% increase 7 days after irradiation of the *het* limb muscle cross-sectional area, significant when compared to non-irradiated *het* limb muscle. However, at all the other post-irradiation time points, the mean cross-sectional areas of the *het* irradiated limb muscles were 17.6 to 31.1% smaller than WT limb muscles from non-irradiated mice (Figure 1A, C, D). Non-irradiated *het* limb muscles, as expected, showed evidence of severe disease and cycles of fiber degeneration and regeneration, with over 58% of their myofibers containing centrally located nuclei (Figure 1B, C, D). However, by 30 days after irradiation, there was a significant reduction in central nucleation compared to the non-irradiated het limb muscles, which was maintained up through 6 months, with a 27.5% decrease at 1 month and a significant 40% decrease by 6 months compared to non-irradiated *het* control limb muscles and a decrease of approximately 45% at 2 and 6 months compared both to the peak at 7 days and at 14 days post-irradiation (Figure 1B, C). This suggested that 18 Gy irradiation had a long term impact on the regenerative cell populations in the irradiated *het* limb muscles.

### Gamma Irradiation of the Wild Type and Het Mice EOM

**Figure 2:**
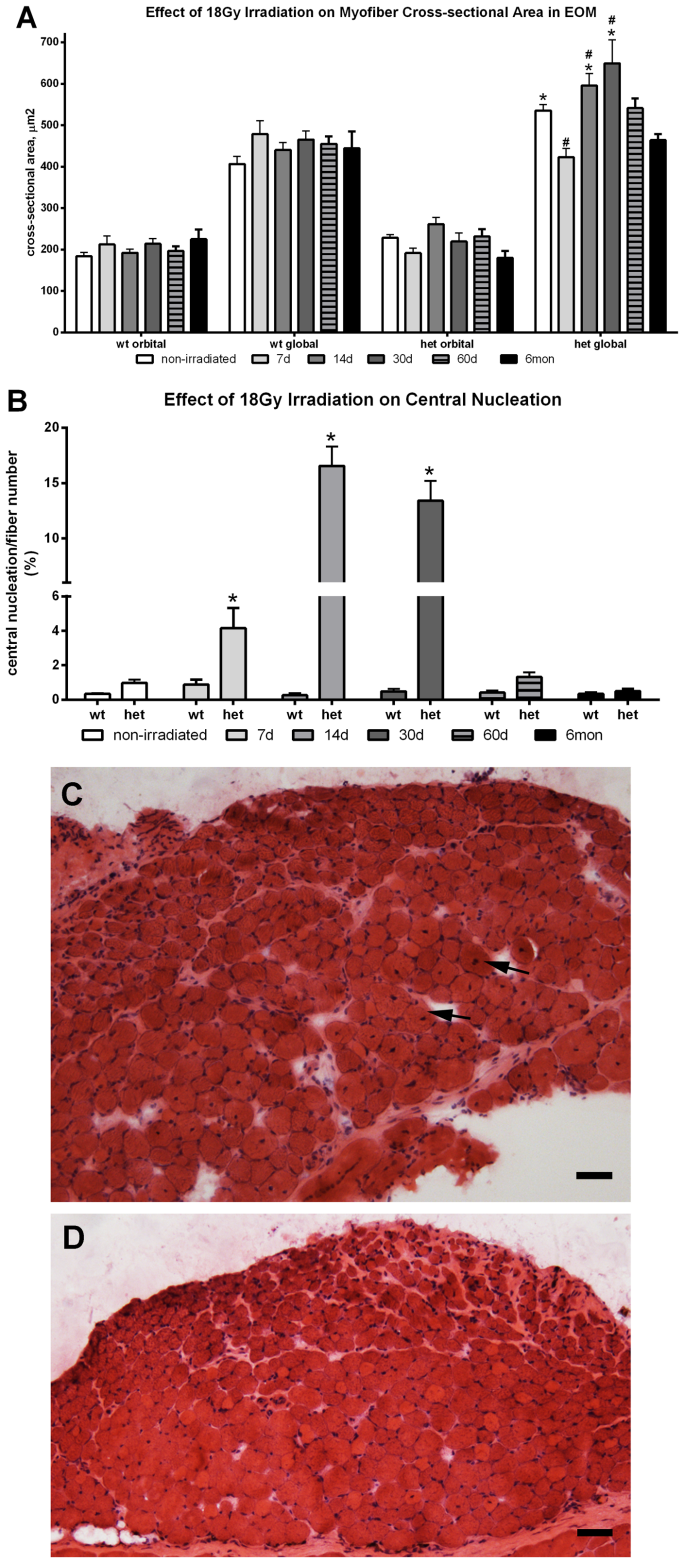
Effect of 18 Gy Irradiation on Cross-sectional Areas and Central Nucleation in Extraocular Muscles from Wild Type and *Het* Mice. A. Effect of 18 Gy irradiation on cross-sectional areas in extraocular muscle (EOM) from WT and *mdx:utrophin^+/−^* mice (*het*) mice. *indicates significant difference from non-irradiated WT control. # indicates significant different from non-irradiated *het* muscle. B. Effect of 18 Gy irradiation on central nucleation in EOM from WT and *het* mice. *indicates significant difference from non-irradiated WT and *het* muscles. C. Photomicrographs of *het* extraocular muscle 14 days after 18 Gy irradiation. Arrows indicate myofibers with centrally located nuclei. D. Wild type (WT) extraocular muscle 14 days after 18 Gy irradiation. Sections are stained with hematoxylin and eosin. Bar is 50 µm.

In the irradiated WT EOM, no inflammatory infiltrate or myofiber necrosis was seen at any of the post-irradiation time points. Irradiated EOM from WT mice did not show significant changes in cross-sectional area after gamma irradiation at any post-irradiation time point (Figure 2A), nor were there any changes to the low rate of central nucleation found in WT EOM (Figure 2B).

As seen in the WT irradiated EOM, no evidence of increased inflammatory infiltrate or widespread myofiber necrosis was seen in the irradiated *het* EOM (Figure 2 C, D). However, in contrast, the mean EOM myofiber cross-sectional areas in the non-irradiated *het* mice were 23% and 29.5% larger than those in WT mice (Figure 2A). In both the orbital and global layers, there was a transient decrease in cross-sectional area at 7 days post-irradiation, 17 and 20% respectively, compared to non-irradiated het EOM, but only significant for the global layer myofibers. However, the mean cross-sectional areas in the *het* EOM at 7 days post-irradiation were not significantly different from WT control values. In the irradiated *het* EOM, this transient decrease was followed by a significant increase in mean myofiber size in the global layer, which was 11.8% and 21.9% greater than non-irradiated *het* muscles at 14 and 30 days post-irradiation. Compared to WT control EOM, this represents a 46.66% and 59.85% increase in mean myofiber cross-sectional area. Thus, in contrast to the limb skeletal muscle myofibers, irradiation resulted in a significant increase in mean myofiber size in the EOM global layer fibers at 14 and 30 days post-irradiation. The myofibers then gradually returned to cross-sectional areas that were not significantly different from non-irradiated *het* EOM over the next few months (Figure 2A). In addition, the *het* EOM maintained cross-sectional areas similar to the WT controls 6 months after irradiation treatment.

Most striking, however, was the significant increase in central nucleation in the irradiated *het* EOM, specifically in the global layer (Figure 2B, C, D). One week after 18 Gy irradiation of the *het* EOM, there was a 4-fold increase in the incidence of central nucleation. By two weeks after irradiation, there was 60-fold increase in the percent of myofibers in the EOM that were centrally nucleated (Figure 2B, C, D), which was maintained at one month post-irradiation with a 50-fold increase compared to *het* non-irradiated EOM. Essentially, at one, two and four weeks after a single 18 Gy dose of gamma irradiation, the EOM acquired a dystrophic phenotype (Figure 2C). Just as striking is the complete return to normalcy – relative to central nucleation – by 2 months and maintained at 6 months. This suggests that there is a dynamic change in the process of myofiber remodeling/repair in the short term after 18 Gy irradiation, which resolves and returns to the normal process of EOM remodeling in these irradiated *het* EOM at 2 and 6 months.

### Effects of Gamma Irradiation on brdU Incorporation into Het EOM and Leg Muscles

**Figure 3:**
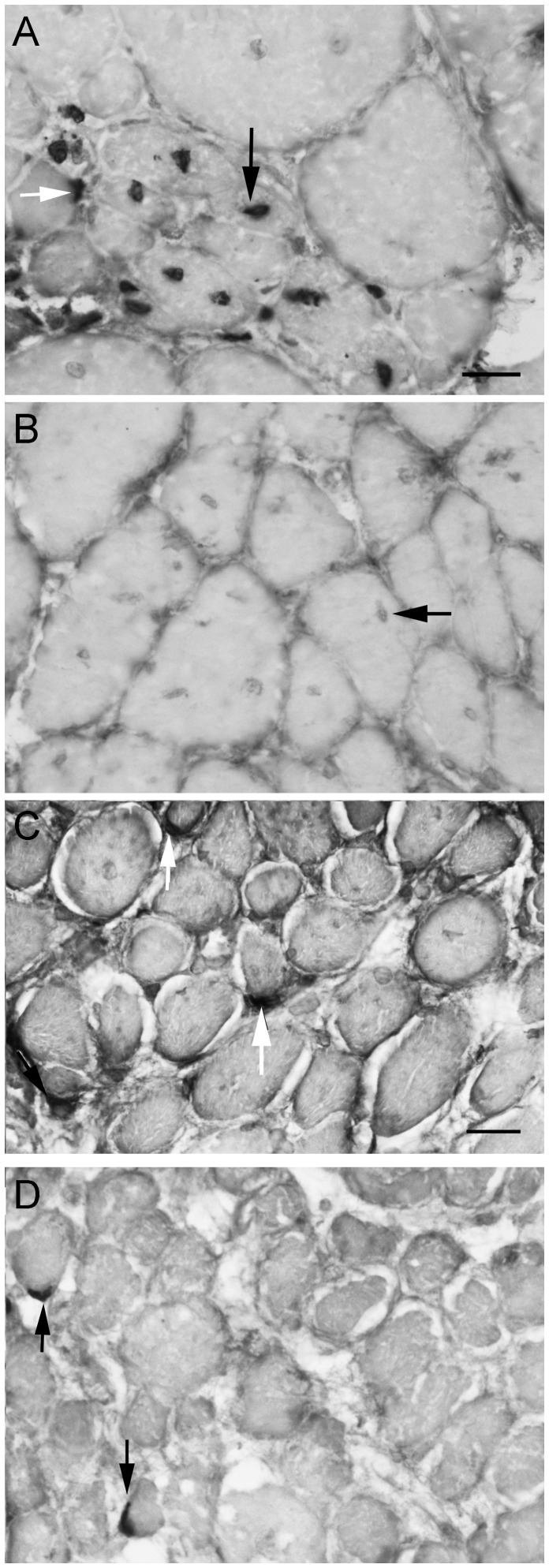
Bromodeoxyuridine Labeling of Irradiated and Non-Irradiated Limb and Extraocular Muscle from *Het* Mice. Photomicrographs of sections from *het* leg muscle and *het* EOM 7 days after 18 Gy irradiation followed by daily bromodeoxyuridine (brdU) injections for 7 days and immunostained for brdU. A. Non-irradiated *het* leg muscle. Arrow indicates a brdU-positive myonucleus. B. Irradiated *het* leg muscle. Horizontal arrow indicates a non-brdU labeled myonucleus. C. Non-irradiated *het* EOM. Cells positive for brdU are indicated by white arrows. D. Irradiated *het* EOM. Cells positive for brdU are indicated by vertical black arrows. All photomicrographs are the same magnification. Bar is 20 µm.

BrdU labeling was examined in both naïve and gamma irradiated EOM and limb muscles from *het* mice to assess its effect on proliferating myogenic precursor cells. In the non-irradiated *het* limb muscles, significant brdU-labeling of nuclei was seen after 7 days of brdU injections (Figure 3A). These brdU-positive nuclei were found both in the satellite cell position (Figure 3A, white arrow) and in foci of recently regenerated myofibers as evidenced by the central location of the brdU-positive nuclei (Figure 3A, black arrow). BrdU-positive nuclei were not found 7 days after irradiation of the *het* limb muscles, which was expected due to the preferential cell death of dividing myogenic precursor cells after gamma irradiation (Figure 3B). Note that faint centrally located nuclei were still present a result of previous regeneration prior to brdU labeling; they were just not labeled with brdU (Figure 3B, black arrow). In contrast, brdU-labeled nuclei in the satellite cell position were present in both the *het* non-irradiated (Figure 3C) and het irradiated (Figure 3D) EOM. This suggested that at least a subset of the myogenic precursor cells in EOM appear to be resistant to 18 Gy irradiation.

### Effect of Gamma Irradiation on Pax7 Cell Density

**Figure 4:**
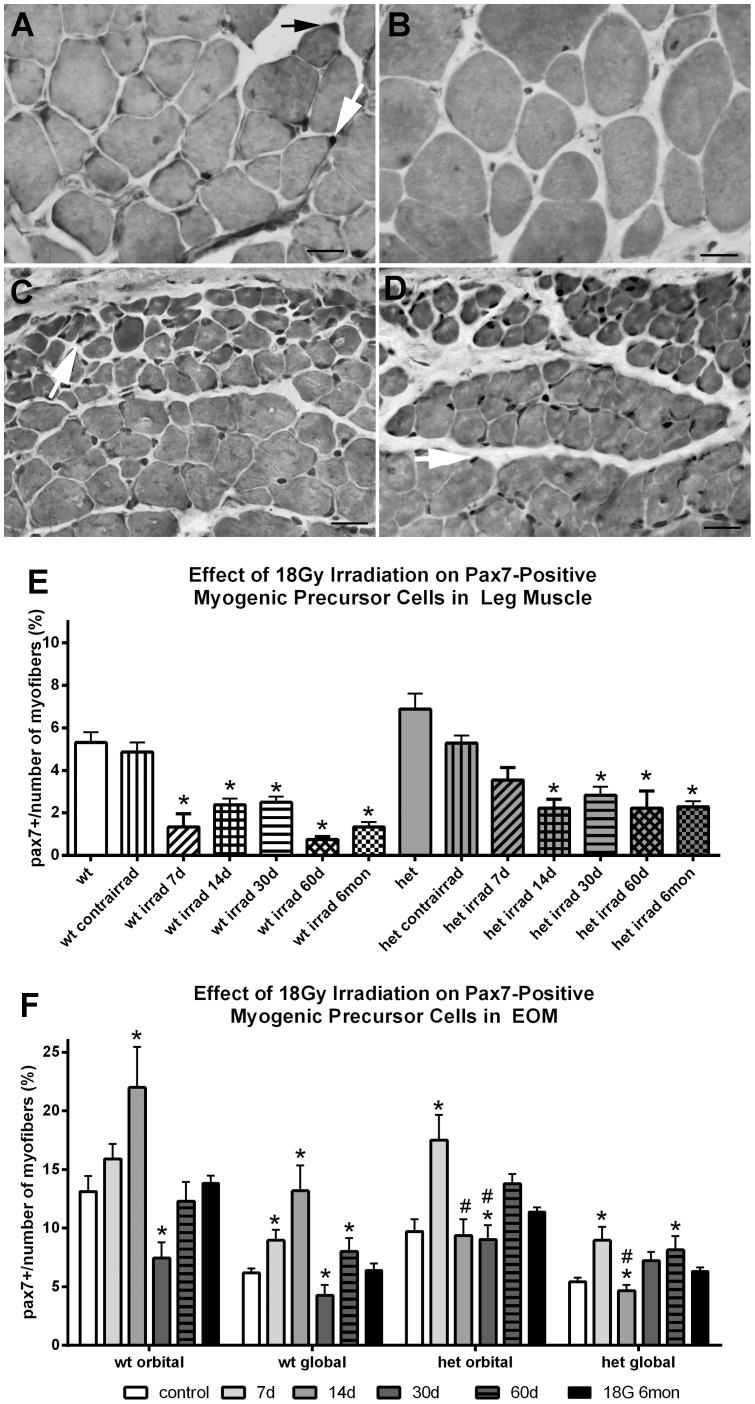
Effect of 18 Gy Irradiation on Pax7 Cell Density in Limb and Extraocular Muscles from Wild Type and *Het* Mice. A. Cross-section of non-irradiated *het* leg muscle immunostained for Pax7-positive cells, indicated by black and white arrows. B. Cross-section of *het* leg muscle 7 days after 18 Gy irradiation and immunostained for the presence of Pax7-positive cells. No positive cells can be seen. C. Cross-section of non-irradiated *het* EOM immunostained for the presence of Pax7-positive cells, an example indicated by white arrow. D. Cross-section of *het* EOM 7 days 7 days after 18 Gy irradiation and immunostained for the presence of Pax7-positive cells. All the black nuclei are Pax7-positive cells. Bar is 20 µm. E. Effect of 18 Gy irradiation on Pax7-positive cell density in leg muscle *indicates significant difference from non-irradiated WT control and non-irradiated *het* control. F. Effect of 18 Gy irradiation on Pax7-positive cell density in EOM from WT and *het* mice. *indicates significant difference from non-irradiated WT control and non-irradiated *het* control. **#** indicates significant difference from non-irradiated WT orbital control.

As a result of this dramatic appearance of a dystrophic phenotype in the irradiated *het* mouse EOM and the reduction in brdU-positive cells after irradiation, analysis of changes to specific populations of myogenic precursor cells was undertaken. Irradiation at 18 Gy resulted in a significant reduction of Pax7-positive cells in both WT and *het* limb muscles, with decreases ranging from 50–85% of the non-irradiated levels (Figure 4A, B, E). This decrease in the Pax7 population mirrored the decline in central nucleation seen after irradiation of the *het* limb muscle.

In contrast to limb muscles, irradiation of WT EOM resulted in a significant increase in Pax7-positive cells in the orbital layer at 14 days and the global layer at 7 and 14 days post-irradiation, 21% and 67.6% for the orbital layer, and 44.8% and 113% for the global layer. This was followed by a significant decrease in the Pax7-positive cells in the WT EOM 30 days post-irradiation, a rebound increase at 2 months, and a return to normalcy by 6 months.

Gamma irradiation also resulted in a significant increase in the density of Pax7-positive cells in *het* EOM at 7 days post-irradiation in both orbital and global layers, increases of 80.5% and 65.9% in the orbital and global layers respectively (Figure 4C,D, F). However, at 14 d and 30 d in the orbital layer and at 14 days in the global layer, Pax7 cell density was significantly decreased compared to normal WT levels, although they were not different from the *het* non-irradiated levels. Immunostaining also revealed that there were Pax7-positive centrally located nuclei within the EOM cross-sections within the first month post-irradiation (not shown). The significant alteration in the Pax7 population of myogenic precursor cells in *het* EOM after irradiation suggests that there were more Pax7-positive cells available for muscle remodeling and repair in the period when central nucleation increases began. The pattern of decrease in the density of the Pax7-positive cells, after a significant elevation, correlated temporally with the significant increases in cross-sectional area and central nucleation. This suggests that the observed decrease in the Pax7-positive cells in the two to four week post-irradiation period may be due to their fusion into EOM myofibers resulting in central nucleation in the myofibers of the irradiated *het* EOM. Pax7-positive central nuclei also support this hypothesis.

### Effect of Gamma Irradiation on EECD34-Positive Cell Density

Our previous studies identified a population of myogenic precursor cells that are elevated in EOM compared to limb skeletal muscle and retained in the EOM of mouse models of DMD. These cells, the EECD334 cells, are positive for CD34 and negative for Sca1, CD31, and CD45 [23], [24]. The effect of 18 Gy irradiation was assessed on EECD34 cells using flow cytometry and analyzed on the basis of percent positive per total live mononuclear cells in both WT and *het* EOM and limb muscles. The percent of EECD34 cells in irradiated WT limb muscles decreased 46% after one week, 62% after two weeks, and then tripled in density by one month. However, the density of these cells in limb muscle is extremely low [23], [24]; thus, it is unclear what role these cells play in repair and regeneration in limb skeletal muscle.

**Figure 5:**
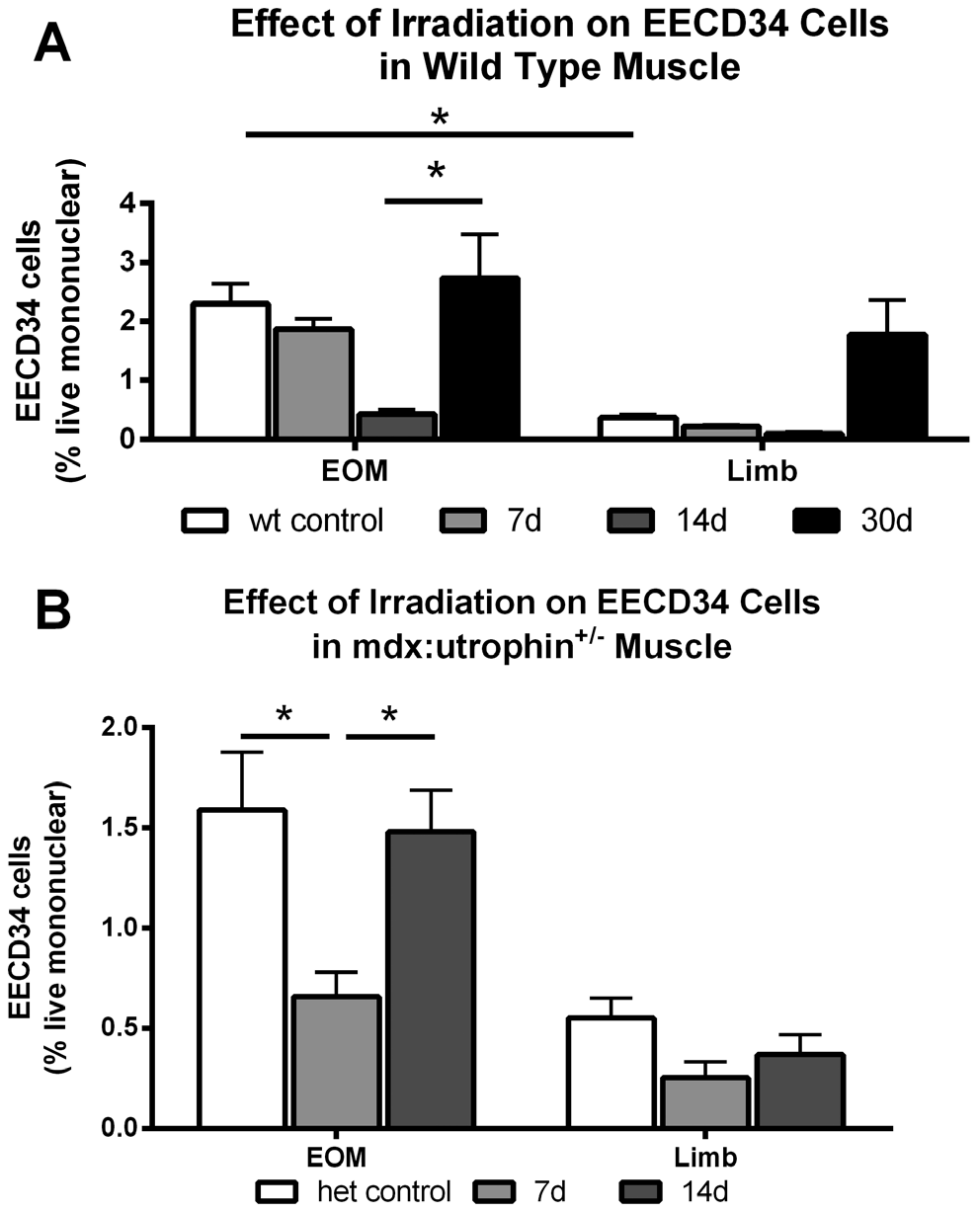
Effect of 18 Gy Irradiation on EECD34 Cell Density in Limb and Extraocular Muscles from Wild Type and *Het* Mice. A. Effect of 18 Gy irradiation on EECD34 cell percentages in WT EOM and limb muscle as determined by flow cytometric analysis calculated based on total live cells. *indicates significant difference. B. Effect of 18 Gy irradiation on EECD34 cell percentages in *het* EOM and limb muscle as determined by flow cytometric analysis based on total live cells. *indicates significant difference.

A similar picture was seen in the EOM of WT mice, where the percent of EECD34 cells stayed constant one week after irradiation, showed a large and significant decrease of 75% two weeks after 18 Gy irradiation, and then returned to normal density by one month (Figure 5A). Thus, while these cells appeared to be initially affected by the 18 Gy irradiation, they rebounded quickly to return to their normal density levels.

The effect of irradiation on the EECD34 cells in the *het* mouse EOM and limb muscles was also examined. The *het* limb muscles showed very little change in their already low levels of these cells. However, the *het* EOM showed a significant decrease of 62.7% in the density of these cells one week after 18 Gy irradiation compared to non-irradiated het EOM, and a return to normal levels by two weeks (Figure 5B). This pattern was similar to the WT EOM changes in the EECD34 cells, but the loss of these cells occurred one week earlier, suggesting that more were proliferating in the *het* EOM and therefore more susceptible to injury from the gamma irradiation. It should be noted, however, that these are relatively rare cells, and most likely represent a “reserve” multipotent precursor cell population [23], [24].

### Effect of Gamma Irradiation on Pitx2-Positive Cell Density

**Figure 6:**
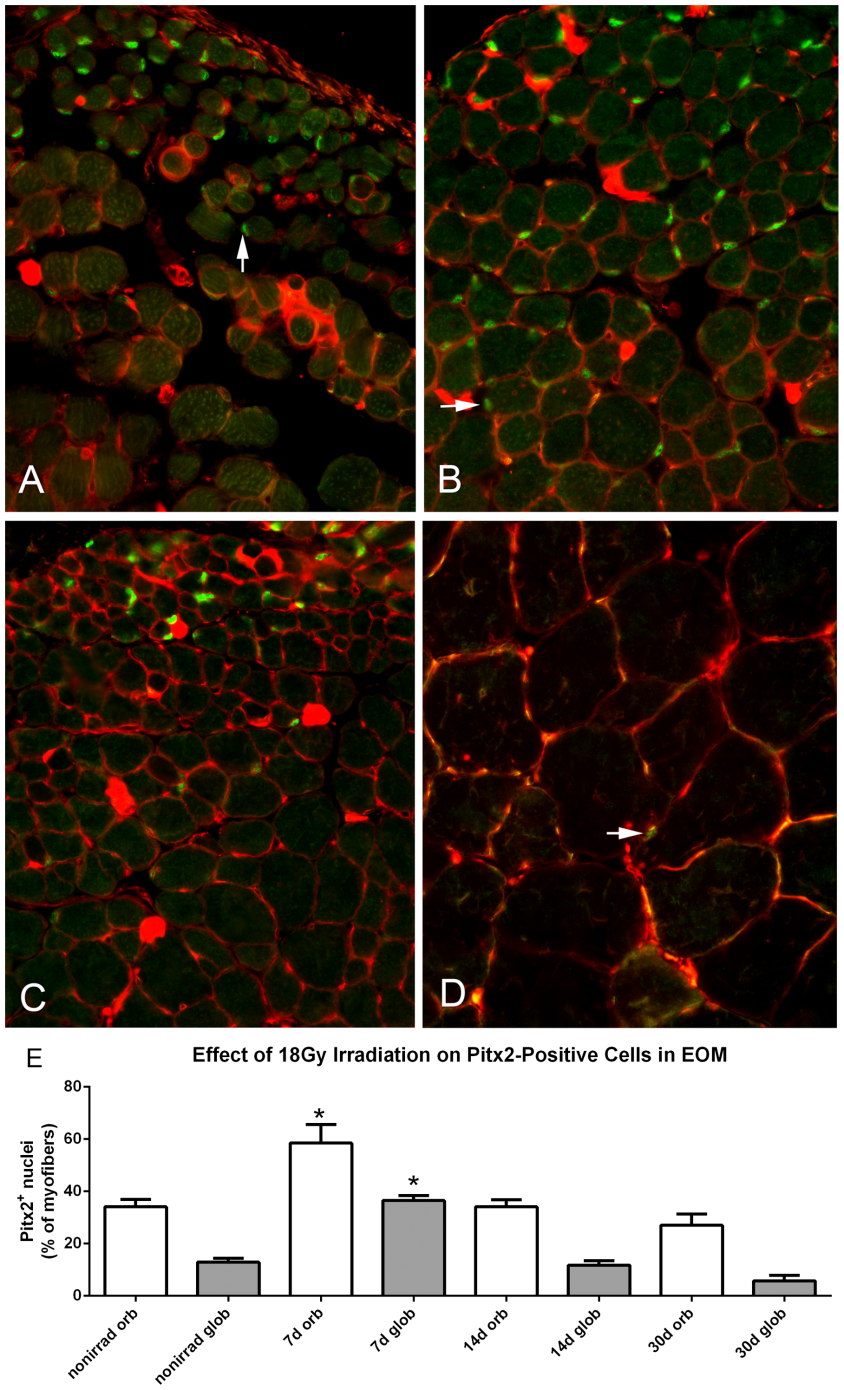
Effect of 18 Gy Irradiation on Pitx2 Cell Density in Extraocular Muscles from *Het* Mice. Photomicrographs of *het* EOM immunostained for Pitx2 (green, indicated by arrows) and wheat germ agglutinin (WGA, red) to visualize the sarcolemma from A. non-irradiated *het* EOM, B. 7 days after 18 Gy irradiation, C. 14 days after 18 Gy irradiation, and D. *het* limb muscle showing the extreme paucity of Pitx2-positive cells. Magnification: x400. E) Morphometric analysis of number of Pitx2 positive cells as percent of myofiber number. *indicates significant difference from non-irradiated *het* control EOM.

**Figure 7:**
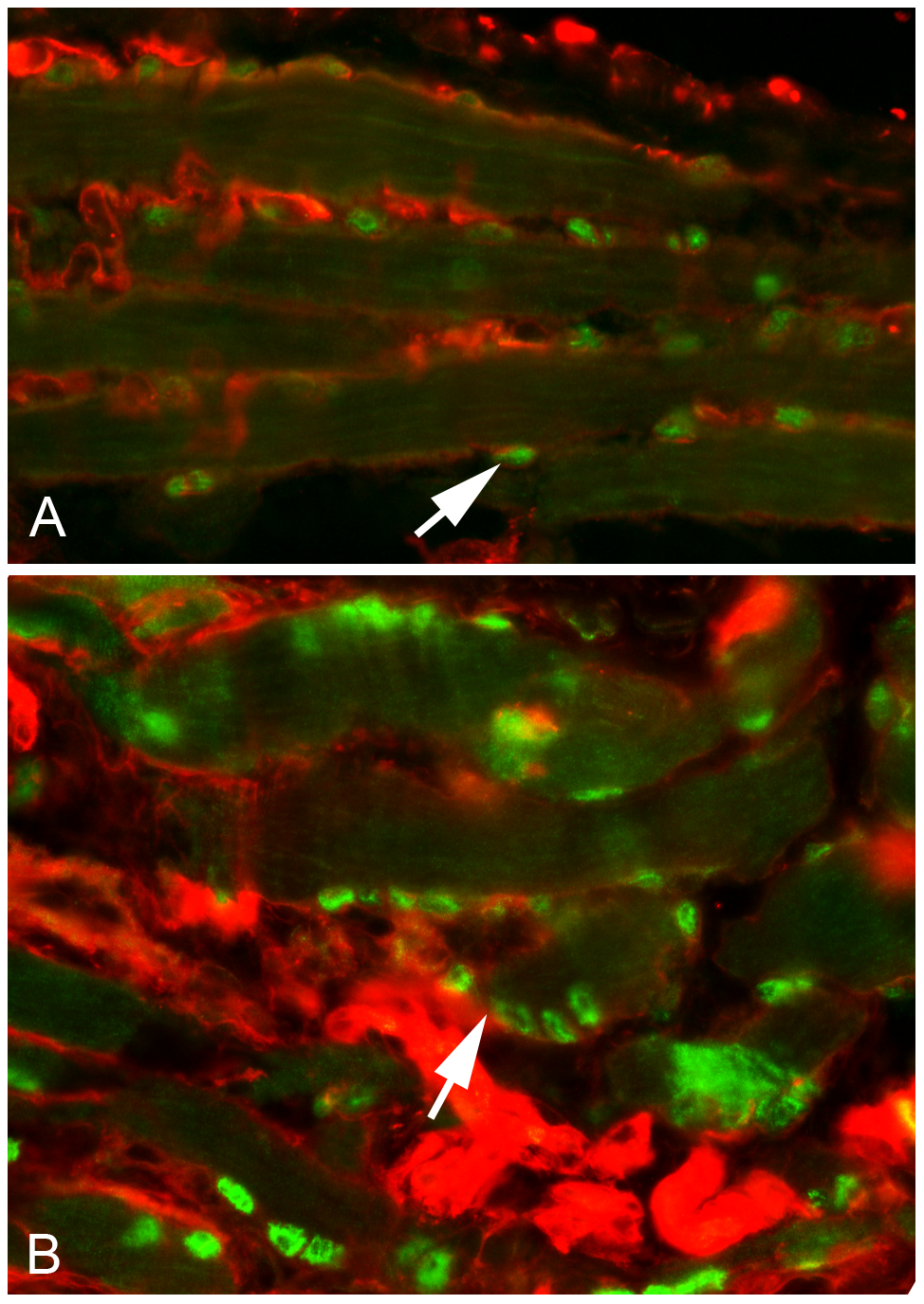
Localization of Pitx2-Positive Nuclei in Extraocular Muscles. A. Photomicrograph of a longitudinal section through a non-irradiated *het* EOM immunostained for Pitx2 (green) and wheat germ agglutinin (WGA, red). Arrow indicates a Pitx2-positive cell outside of the myofiber sarcolemma (red). B. Photomicrograph of a longitudinal section through a *het* EOM 7 days after 18 Gy irradiation. Note the increased number of Pits2-positive cells both in the satellite cell position within the basal lamina of individual myofibers and their location directly deep to the sarcolemma as immunostained by Pitx2 (green) and wheat germ agglutinin (red). An example is indicated by the white arrow. Magnification: x400.

There is another relatively unique population of myogenic precursor cells that express Pitx2, and these are significantly elevated in EOM compared to limb skeletal muscle [24]. While the majority of EECD34 cells also express the transcription factor Pitx2 [24], there are many more Pitx2-positive cells in EOM beyond those that are CD34^+^. These were plentiful in both WT and *het* adult EOM (Figure 6, 7). This population was quantified in tissue sections from irradiated and non-irradiated *het* EOM. Gamma irradiation resulted in an increased density of these cells in both orbital and global layers within one week, with increases of 71.66% and 185.2% respectively. This increased density was particularly large in the global layer where they are normally more sparsely localized (Figure 6A, B, E). The density of Pitx2-positive cells returned to control levels by 14 days post-irradiation (Figure 6E). It should be noted that the Pitx2-positive nuclei were located both external and internal to the WGA membrane staining (red), and no Pitx2-positive nuclei were found centrally located within the myofiber cross-sections (Figures 6, 7).

**Figure 8:**
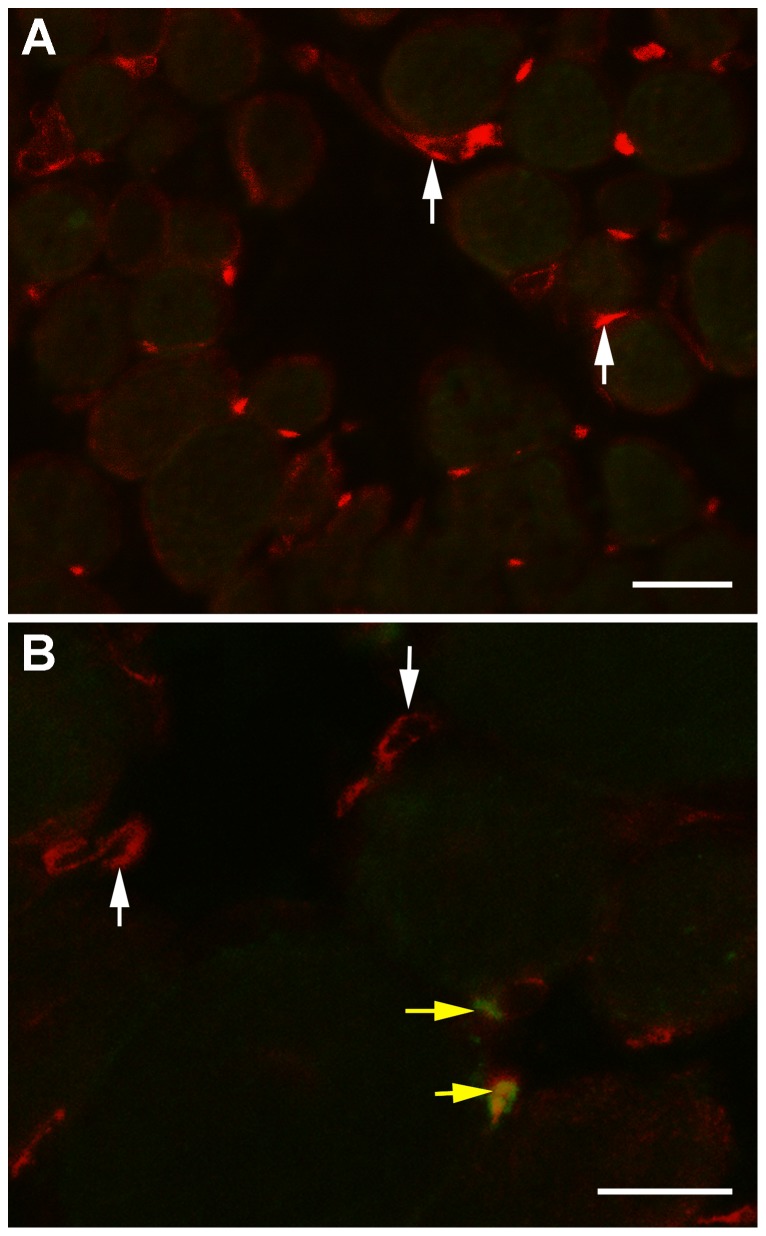
Co-expression of Pitx2 and brdU in Extraocular Muscles from *Het* Mice. A. The vast majority of Pitx2-positive cells in both irradiated (shown) and non-irradiated (not shown) *het* EOM were brdU-negative (vertical white arrows) B. Although rare, brdU-positive cells that co-expressed Pitx2 were also seen in the irradiated *het* EOM (horizontal yellow arrows). Bar is 20 µm.

To determine if the increase in Pitx2-positive cells at 7 days post-irradiation was due to replication or differentiation of existing precursor cells, *het* EOM cross-sections immunostained for both Pitx2 and brdU were examined. The vast majority of the Pitx2-positive cells 17 days after the 18 Gy irradiation were brdU-negative (Figure 8A). In the non-irradiated (shown) and irradiated EOM, cells positive for brdU and Pitx2 were observed; however, they were relatively rare (Figure 8B).

### Effect of Irradiation on Collagen I and IV

**Figure 9:**
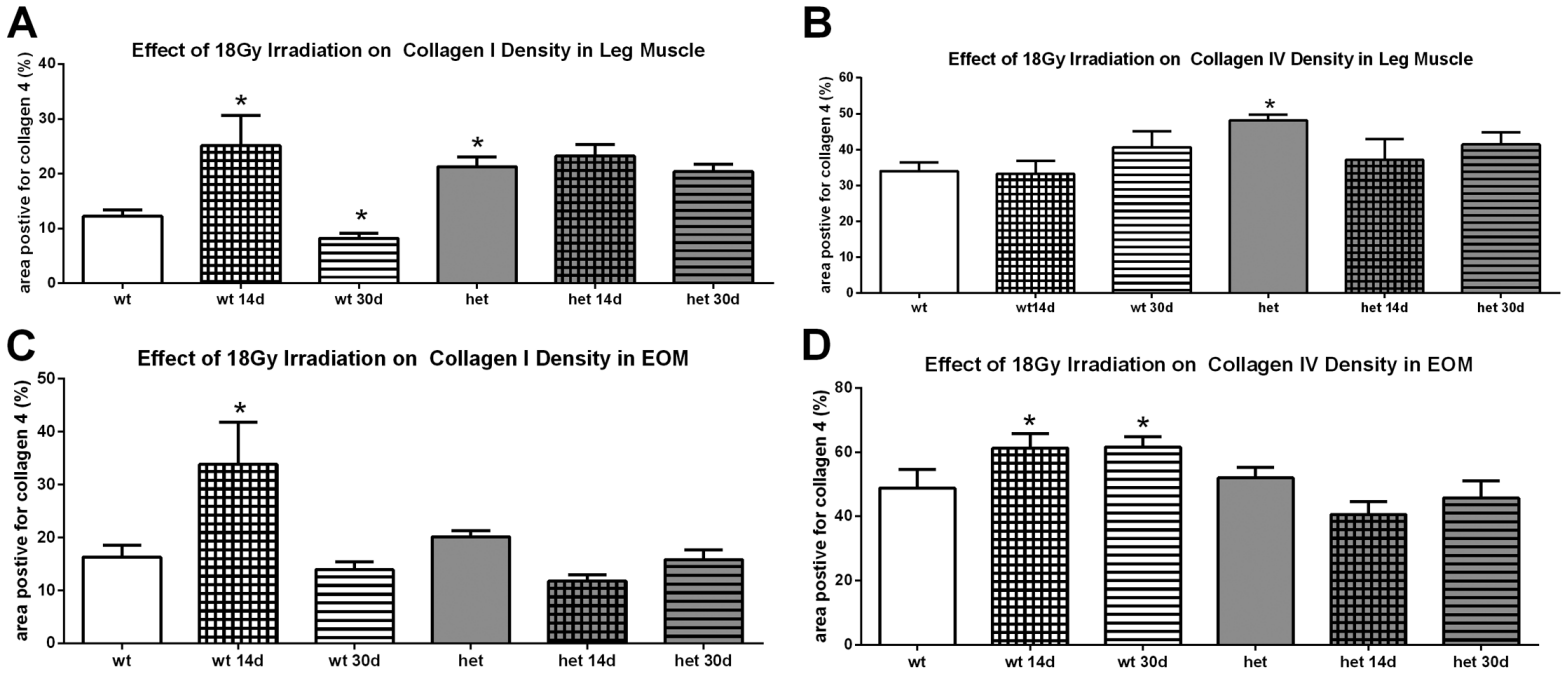
Effect of 18 Gy Irradiation on Collagen Density in Limb and Extraocular Muscles from Wild Type and *Het* Mice. A. Effect of 18 Gy irradiation on collagen I levels in WT and *het* leg muscle. B. Effect of 18 Gy irradiation on collagen IV levels in WT and *het* leg muscle. C. Effect of 18 Gy irradiation on collagen I levels in WT and *het* EOM. D. Effect of 18 Gy irradiation on collagen IV levels in WT and *het* EOM. *indicates significant difference from non-irradiated WT muscle.

It is possible that the development of a dystrophic pathology in the *het* EOM was due to the effects of irradiation on connective tissue elements and resultant fibrosis. The levels of both collagen I and collagen IV were determined in these muscles and in the limb muscles. In limb muscles, the *het* mice had a significantly greater density of both collagens when compared to the WT mouse muscles, 73.9% more collagen I and 41.5% more collagen IV (Figure 9). Irradiation resulted in a 100% increase in collagen I in the WT limb muscles 14 days after treatment, and this was followed by a return to non-irradiated levels by 30 days (Figure 9A). Irradiation did not result in a change in collagen I in the *het* limb muscles compared to non-irradiated *het* limb muscles. Irradiation resulted in no change of collagen IV density in WT or *het* limb muscles (Figure 9B).

There was no statistically significant difference in collagen I or IV density between the non-irradiated WT and *het* EOM. In WT mice, irradiation resulted in collagen I changes in WT EOM similar to those seen in limb muscles, with a 100% increase in collagen I density 2 weeks after irradiation followed by a return to normal non-irradiated collagen I levels (Figure 9C). In the WT EOM, irradiation produced a 25.4% increase in collagen IV at 2 and 4 weeks after irradiation of WT EOM. Interestingly, irradiation of *het* EOM did not result in any significant changes in the density of either collagen I or IV (Figure 9).

## Discussion

Gamma irradiation of 18 Gy had little effect on WT limb or EOM in the short or long term; there were few changes in muscle fiber size or central nucleation. In contrast, the irradiated *het* limb muscle showed a decrease in both muscle fiber size and central nucleation that was mirrored by a decline in the number of resident Pax7-positive satellite cells in the irradiated limb muscles. This suggests that in the *het* mice gamma irradiation reduced the population of Pax7 satellite cells to a level that resulted in insufficient numbers in the dystrophic mouse limb muscles to maintain their muscle mass with increased post-irradiation duration. The EOM response to 18 Gy irradiation was quite different. First, non-irradiated *het* EOM myofibers were significantly larger in mean cross-sectional area than WT EOM myofibers. Gamma irradiation of *het* EOM caused a short-term decrease in myofiber size at 7 days post-irradiation. However, in stark contrast to *het* limb muscle, at 14 and 30 days post-irradiation the *het* EOM myofibers were significantly larger than the non-irradiated *het* EOM myofibers. The myofibers in the *het* EOM gradually returned to normal *het* cross-sectional areas by 2 months post-irradiation. Most provocative was the appearance of a dystrophic phenotype, as defined by central nucleation, in the *het* EOM in the month following irradiation. We hypothesize that the EOM actively maintain their normal morphology in muscular dystrophies; irradiation removes one or more populations of myogenic precursor cells whose function is taken over by a distinctly different precursor type whose fusion into existing myofibers results in central nucleation.

### Post-Irradiation: Limb Muscle

The effects of gamma irradiation on limb muscle were as predicted. In agreement with previous studies, analysis showed that high doses of gamma irradiation have little effect on WT limb muscle fiber size [41]. The reduction in central nucleation and mean myofiber cross-sectional area in *het* triceps muscles after irradiation agrees with previous studies in irradiated soleus and extensor digitorum longus muscles of adult *mdx* mice [25], [28], [30], [40]. Additionally we showed a clear temporal correlation between the *het* limb muscle reductions in myofiber size and central nucleation and the density of Pax7-positive myogenic precursor cells. While a similar significant reduction in Pax7-positive cell density was seen in the WT irradiated limb muscles, the lack of injury or disease in these muscles presumably protected them from this loss of satellite cells. Injury to these irradiated WT limb muscles may be able to demonstrate deficiencies in the regenerative potential caused by the significant loss of Pax7-positive satellite cells, as would be predicted by the literature [31], [42], [43], [44].

### Post-Irradiation: Het EOM at 7 Days

Similar to WT limb muscle, 18 Gy irradiation did not cause a change in EOM myofiber size in the WT mice. Irradiation of *het* EOM resulted in a short term reduction in mean cross-sectional area in the EOM. This change correlated temporally with significant increases in Pax7-positive cells and Pitx2-positive cells. Our previous study demonstrated that there is little to no overlap between these two different myogenic precursor cell populations in EOM [24]. The initial four-fold increase in central nucleation in the irradiated *het* EOM at 7 days post-irradiation coincided temporally with the large elevation in Pax7-positive cells. As these muscles had been irradiated, and the brdU analysis showed a decrease in brdU-positive satellite cells, it is presumed that this increase is due to differentiation of Pax7-positive cells from pre-existing precursor cells residing in the EOM. This is supported by studies demonstrating that muscle hypertrophy induced by stretch or mechanical overload can occur, with concomitant up-regulation of MyoD and myogenin in the absence of satellite cell proliferation [45], [46]. While skeletal muscles with tamoxifen-induced satellite cell depletion were regeneration-deficient, the muscles of those Pax7-depleted muscles retained the ability to hypertrophy, that is increase their cross-sectional areas, after unloading-induced atrophy [47]. While increased central nucleation at 7 days post-irradiation correlated temporally with increased density of Pax7-positive cells, it also correlated with decreased myofiber cross-sectional areas. This seems counterintuitive in light of the fact that Pitx2-positive cells were also increased in density. However, several potential mechanisms can be evoked. First, only an extremely rare Pitx2-positive cell was also brdU positive, suggesting that their up-regulation in density was the result of differentiation of a precursor cell that is of yet uncharacterized. In normal EOM, 80% of EECD34 cells were Pitx2-positive, while many Pitx2-positive cells were CD34-negative [24]. One hypothesis is that the EECD34 cells (or some other yet undefined cell type) are precursor cells to the Pitx2-positive population; this would explain the decrease in EECD34 cells and the concomitant increase in Pitx2-positive cells in the absence of proliferation. As the number of centrally nucleated myofibers is lower at 7 days post-irradiation than at 14 days post-irradiation, it may be that at shorter time intervals, these cells would be reduced in density compared to non-irradiated *het* EOM. Thus, the elevated numbers are a response to some signal from the irradiated, and presumably dying proliferative cells as a result of irradiation, and this in turn would initiate their rapid replenishment. In addition, the majority of Pitx2-positive nuclei in normal EOM are peripherally located myonuclei [24], [47], [48], [49]. In contrast, it appears that many of the Pitx2-positive nuclei in the *het* irradiated EOM appeared to be in the satellite cell position. We suggest, and these results support, the hypothesis that Pitx2-positive myogenic precursor cells maintain EOM cross-sectional area, but in contrast to Pax7-positive myogenic precursor cells, they fuse with the EOM myofibers and maintain peripheral nucleation, as central nucleation and increased myofiber cross-sectional areas in the *het* irradiated EOM followed periods of increased density of both Pax7 and Pitx2-positive myogenic precursor cells. (See Figure 7).

### Post-Irradiation: Het EOM at 14 and 30 Days

The het EOM myofibers were significantly larger than those in the non-irradiated *het* EOM. The concomitant appearance of large numbers of centrally nucleated myofibers in the irradiated *het* EOM together with increased myofiber sizes at 2 and 4 weeks after irradiation suggests that irradiation significantly altered the normal process of EOM remodeling in *het* EOM that is normally involved in their sparing [18], [20]. There are a number of examples in the literature where the EOM respond to perturbations or disease in a direction opposite to that of limb skeletal muscles. For example, after treatment with botulinum toxin A, limb skeletal muscle fibers atrophied during the period of chemical denervation [50], [51]. After treatment of the EOM with botulinum toxin, the myofibers actually became larger in mean cross-sectional area [22], [52]. In addition to their sparing in DMD, the EOM are also spared in the sarcopenia associated with aging and in neurogenic wasting diseases such as amyotrophic lateral sclerosis [53], [54]. What is not seen in the EOM of humans or animals with these diseases or after injury is the appearance of central nucleation.

This increased central nucleation and the increased myofiber areas correlated temporally with a return to the normal density of the Pax7- and Pitx2-positive cells. Fusion of a large number of Pax7-positive satellite cells into the het EOM myofibers would also explain the resulting drop in Pax7-positive cell density seen at 14 days post-irradiation. However, it is also quite interesting that in contrast to *het* limb muscle, 18 Gy irradiation did not result in the long-term reduction in density of the Pax7-positive cell population. This dynamic capacity to respond with increased proliferation in the Pax7-positive satellite cell population in EOM has been seen in previous studies involving EOM perturbations including chemical denervation and surgery [22], [55]. A number of factors could explain this difference between what are ostensibly the same cells in EOM and limb skeletal muscle. First, the precursor stem cell in the EOM for the Pax7-population may be more resistant to irradiation-induced injury due to the presence of protective factors present in EOM but either absent or at insufficient levels in limb skeletal muscle. For example, the EOM maintain high levels of insulin-like growth factor-I (IGF-I) and its receptor [56], [57], [58], which has been shown to have protective effects on myogenic precursor survival [59], [60]. Another possibility is that the myogenic precursor cells, or a subset of them, are less susceptible to injury based on some intrinsic property as yet undefined. We have demonstrated resistance of the EECD34 cells to oxidative stress in vitro [23] and increased survival of myogenic precursor cells even in procedures used to isolate them [61].

The Pitx2 population returns to normal levels 2 weeks post-irradiation in the *het* EOM. As knock-down of Pitx2 results in a decreased fusion index *in vitro* [24], we hypothesize that Pitx2-positive myogenic precursor cells in EOM fuse continuously into existing uninjured EOM myofibers and maintain peripheral nucleation during this process. The initial increase and sustained maintenance of Pitx2-positive cells suggests that both the Pitx2-positive cells and the precursor cells for the Pitx2-positive cells are resistant to 18 Gy irradiation, and respond to gamma irradiation by increased differentiation into Pitx2-positive cells. There is certainly precedence for a myogenic precursor cell type that is resistant to irradiation at 18 Gy [32]. In contrast to what we see in the EOM of the *het* mice in our study, the irradiation resistant myogenic stem cells seen in notexin-injured muscle in normal mice were significantly diminished in the notexin-injured irradiated *mdx* mice [32]. These different results support our working hypothesis that the EOM have myogenic precursor cell populations with markedly different properties of survivability and proliferation rates than those limb skeletal muscle, even if identified with the same cell markers (e.g. Pax7). This is supported by our *in vitro* studies of EECD34 cell proliferation, which is different in cells derived from EOM as compared to limb muscle [24].

### Working Model

**Figure 10:**
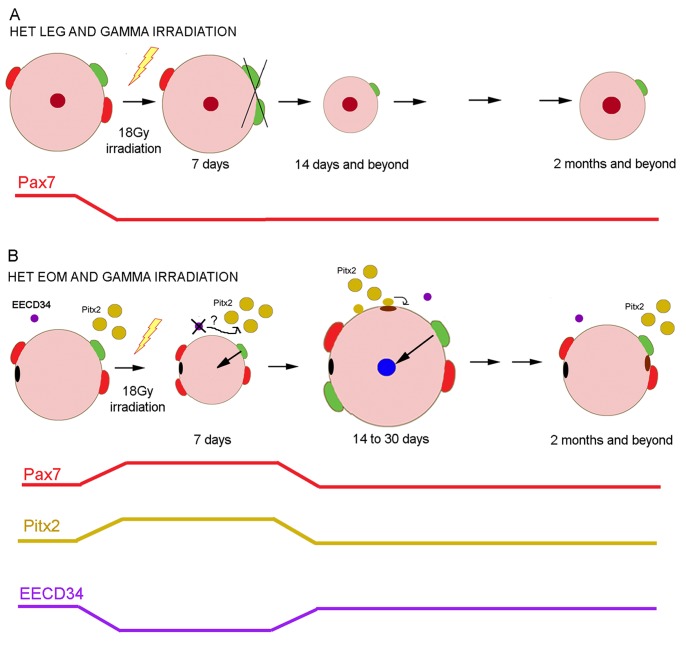
Model of Our Working Hypothesis of the Effect of 18 Gy Irradiation on *het* Mouse Limb and Extraocular Muscles. A. In *het* limb skeletal muscle, gamma irradiation (lightning bolt) damages the Pax7-positive myogenic precursor pool, reducing Pax7 cell density (shown by red line), which in turn ultimately results in smaller myofibers and less central nucleation. Line at the bottom summarizes the change in Pax7-positive myogenic precursor cell density post-irradiation. B. In *het* EOM at 7 days post-irradiation, there is an onset of central nucleation and a reduction in myofiber cross-sectional area. This correlates temporally with increases in Pax7 and Pitx2 cell density and decreases in EECD34 cells. At 14 days post-irradiation, central nucleation is wide-spread in the global layer, myofiber areas increase, and the density of all myogenic precursor cells studied returns to normal. We hypothesize that fusion of the Pax7-positive cells into individual myofibers results in central nucleation, supported by finding Pax7-positive central nuclei, while the Pitx2-positive cells fuse into the existing myofibers but remain peripherally located. As time progresses, the normal equilibrium in the process of EOM myofiber remodeling returns, with a return to normal myofiber size, peripheral nucleation, and myogenic precursor cell density. Red: Pax7-positive cells. Green: activated satellite cells. Mustard: Pitx2-positive cells. Purple: EECD34 cells. Blue: newly added centrally located nuclei. Maroon: original centrally located nuclei. Brown: newly added peripherally located nuclei. Black: original peripherally located nuclei.

We propose that in *het* limb muscle, 18 Gy irradiation markedly decreases the population of Pax7 satellite cells, and over time this results in less central nucleation (Figure 10A). We also propose that *het* EOM maintains the continuous remodeling seen in normal EOM [20]. In *het* EOM, at 7 days post-irradiation the decreased myofiber cross-sectional area and the initial increase in central nucleation correlate temporally with increases in the Pax7 and Pitx2-positive precursor cell densities and a concomitant decrease in the EECD34 cell density (Figure 10B). As there is little evidence that the increased numbers of these precursor cells are due to proliferation at these shorter time points, we suggest that the increased density is due to their differentiation from a radiation-resistant precursor cell population. We hypothesize that at 14 days post-irradiation, the Pax7-positive cell density decreases as a result of their fusion into EOM myofibers forming centrally located myonuclei; the Pitx2-positive cells return to their normal function, where their fusion into EOM myofibers results in Pitx2-positive myonuclei that are peripherally located (Figure 6) and resulting in significantly increased myofiber cross-sectional areas. Thus, concurrently there is Pax7 and Pitx2-positive cell fusion with existing myofibers in the irradiated *het* EOM by 14 days post-irradiation (Figure 10B). By two months, both populations have returned to their normal densities in the *het* EOM, and normal appearance is regained. On-going studies are testing this hypothesis by examination of shorter time points after gamma irradiation.

### Post-Irradiation: Collagen I and IV

Collagens are a large super-family of proteins, but they can be grouped by function [62], [63]. Collagen I is one of predominant forms found in skeletal muscle, in the group of fibril-forming collagens, and is involved in providing tensile strength and rigidity to muscles. In comparison, collagen IV is the major basement membrane collagen of the body and surrounds myofibers, providing a collagenous skeleton within skeletal muscles. We have shown that both of these are expressed in the EOM [64].

While the densities of collagens I and IV were significantly elevated in *het* limb muscle, there was no difference between the densities of collagen I and IV in WT or *het* EOM. This has been reported for limb muscle in *het* mice, but the EOM have not been studied previously [33], [65]. It is quite interesting that irradiation at 18 Gy did not significantly alter the overall levels of either of these collagens in the EOM or limb muscles. While it has been reported that 20 Gy irradiation resulted in a significant loss in fibrosis in *mdx* mice, this study was performed in 10 day old mice in a period when they are undergoing rapid muscle growth [25] as opposed to adult mice in the present study. Our results are supported by an *in vitro* study showing that this dose of gamma irradiation preferentially hampered fibroblast replication and growth, which resulted in enrichment of terminally differentiated myofibers in their cultures [66]. Based on our results, it does not appear that irradiation of either EOM or limb muscles changes levels of tissue fibrosis, at least as measured by collagen I and IV levels.

### Conclusions

We hypothesize that the EOM is spared in muscular dystrophy due to a population of myogenic precursor cells that is either highly proliferative or better able to survive in the diseased environment of a dystrophic muscle. The experiments described in this study support this hypothesis, as alterations in myogenic precursor cell proliferation presumably caused by 18 Gy irradiation resulted in altered levels of specific subpopulations of myogenic precursor cells and the appearance of a dystrophic phenotype in the EOM of the *het* (*mdx:utrophin^+/−^)* model of muscular dystrophy [33], [65]. We have correlated this temporally with up-regulation of Pax7 and Pitx2 myogenic precursor cells and the down-regulation of EECD34 cells. Even though identified using the same cellular markers, e.g. Pax7, it is clear that the ability to survive gamma irradiation differs between the *het* EOM and limb muscle. On-going studies are working to define more completely the molecular and functional differences between these myogenic precursor cells in EOM that normally allows for their complete sparing in DMD. By understanding why the EOM are spared in DMD and other forms of muscular dystrophy, ultimately we hope to elucidate the molecular pathway(s) that control the sparing of the myogenic precursor cells in EOM, which in turn may provide a therapeutic target for enhancing the regenerative capacity and regenerative duration of the myogenic precursor cells in dystrophic limb skeletal muscle.
